# Topical Plant Polyphenols Prevent Type I Interferon Signaling in the Skin and Suppress Contact Hypersensitivity

**DOI:** 10.3390/ijms19092652

**Published:** 2018-09-06

**Authors:** Maria Luigia Carbone, Daniela Lulli, Francesca Passarelli, Saveria Pastore

**Affiliations:** 1 Laboratory of Experimental Immunology, IDI-IRCCS, 00167 Rome, Italy; marialuigia.carbone@idi.it (M.L.C.); d.lulli@idi.it (D.L.); 2 Pathology Unit, IDI-IRCCS, 00167 Rome, Italy; f.passarelli@idi.it

**Keywords:** EGFR, cetuximab, gefitinib, interferon κ, CXCL10, CCL2, resveratrol, quercetin

## Abstract

Human keratinocytes were recently shown to respond to anti-EGFR (epidermal growth factor receptor) drugs with activation of an interferon-κ-driven autocrine loop, leading to enhanced expression of innate antiviral effectors and of the pro-inflammatory chemokines CXCL10 (C-X-C motif chemokine 10) and CCL2 (C-C motif ligand 2). Here we showed active type I interferon signaling in the skin lesions of cancer patients undergoing treatment with the anti-EGFR drug cetuximab. Strong nuclear positivity for Interferon Regulatory Factor 1 and phosphorylated Signal Transducer and Activator of Transcription 1, enhanced interferon-κ expression and CXCL10 was associated to the epidermal compartment. Notably, 50 micromolar resveratrol and quercetin fully suppressed the low constitutive levels of type I interferon signaling and prevented its activation by the anti-EGFR cetuximab or gefitinib in cultured keratinocytes. In sensitized mice undergoing DNFB (2,4-dinitro-1-fluorobenzene)-induced contact hypersensitivity, local administration of gefitinib prior to elicitation further amplified hapten-induced type I interferon activation, tissue edema, and infiltration by T cells, whereas resveratrol or quercetin suppressed this inflammatory cascade. Overall, these data suggest that topical application of resveratrol or quercetin could be potentially effective in preventing pathological conditions due to overactivation of type I IFN (interferon)-driven circuits in the skin, including the inflammatory manifestations of anti-EGFR drug-induced skin-targeted toxicity.

## 1. Introduction

Interferons (IFNs) are pleiotropic, functionally related cytokines with a central role in the cellular antiviral defense [1]. They possess strong antiproliferative and immunomodulatory properties, and are deeply involved in the activation of innate and adaptive immune responses [2]. Type I IFNs are a family of related glycoproteins with distinct tissue distribution and functions in mammals [3]. Among them, IFN-κ was originally identified as a keratinocyte-specific cytokine [4], although it has been later detected also in monocytes/dendritic cells infiltrating chronic inflammatory skin lesions [5]. In the last years, we have been involved in the effort of tracing the molecular pathways underlying the inflammatory component of skin-directed toxicity of anti-EGFR (epidermal growth factor receptor) drugs, including the monoclonal antibody cetuximab and the small-molecule inhibitors erlotinib and gefitinib [6]. We found that human keratinocytes responded to EGFR blockade with upregulation of a cluster of genes in the type I IFN signature, including the transcription factors IRF1 (interferon regulatory factor 1) and STAT1 (signal transducer and activator of transcription 1), and IFN-κ [7]. More recently, we found the same signature in human keratinocytes treated with trametinib and cobimetinib, two drugs acting as selective inhibitors of the MEK (mitogen-activated protein kinase kinases) 1 and 2 [8], downstream of EGFR-driven signal transduction. Mechanistically, early IRF1 upregulation initiated the type I IFN cascade, including enhanced IFN-κ expression. In its turn, via STAT-1 phosphorylation, IFN-κ receptor signaling further amplified the whole cascade, with enhanced expression of IRF1, a conspicuous number of antiviral effectors and a small cluster of pro-inflammatory mediators mainly comprising the chemokines CXCL10 and CCL2 [7]. Upregulation of these two chemokines is a common finding in inflammatory skin conditions where they promote massive T cell and monocyte recruitment, as previously confirmed in mouse models of DNFB (2,4-dinitro-1-fluorobenzene)-induced irritant and allergic contact hypersensitivity, with further aggravation due to concomitant EGFR pharmacological blockade [9,10].

Intracellular reduction/oxidation status is a key element in the regulation of several signal transduction cascades [11]. Intracellular accumulation of reactive oxygen species is essential also for the activation of type I IFN-driven signaling pathways [12,13], through multiple mechanisms currently under active investigation [14,15]. Notably, independent reports document a condition of oxidative stress in cells treated with EGFR blockers [16]. In particular, treatment with the monoclonal antibody cetuximab was shown to downregulate a complex of cell cytoplasmic and membrane proteins including the glutamine receptor, eventually leading to a decrease of reactive oxygen species-reducing capacity of the cell [17]. Furthermore, increased generation of reactive oxygen species and subsequent mitochondrial dysfunction were found in cells treated with the EGFR-selective inhibitor gefitinib [18]. Of relevance, the small-molecule tyrosine kinase inhibitors, which include gefitinib and erlotinib, activate cytochrome P450-dependent drug biotransformation pathways that in their turn establish a condition of oxidative stress, as we previously discussed [7]. Enhanced expression of reactive oxygen species is nonetheless considered a relevant component of the cytotoxic and proapoptotic effects of all anti-EGFR drugs [16,17,18]. Due to their marked antioxidant capacity and high cell membrane permeability, plant polyphenols were shown to interfere with all major signal transduction systems in a variety of mammalian cell types, and are thus considered as potentially effective anti-inflammatory agents [19,20]. Among plant polyphenols, resveratrol and quercetin can prevent activation of the transcription factors NF-κB (nuclear factor κ light chain enhancer of activated B cells) and AhR, and also of EGFR-extracellular signal-regulated kinases 1 and 2 pathway in human keratinocytes, eventually opposing their pro-inflammatory activation by a variety of stimuli that comprised UVA (ultraviolet A), UVB, lipopolysaccharide, TNF-α (tumor necrosis factor α) and IFN-γ [21,22]. Independent groups also demonstrated that resveratrol and quercetin were effective inhibitors of IRF1 and STAT activation [23,24,25]. Notably, a whole-genome transcript profiling identified IRF1 as the only significantly upregulated IRF as soon as 8 h after hapten application on the skin of rats undergoing DNFB-induced contact hypersensitivity [26]. In the same experimental context, STAT1 was among the most upregulated genes 24 h post-elicitation [26]. Furthermore, topical administration of a STAT1 decoy oligonucleotide prior to contact hypersensitivity elicitation attenuated the subsequent inflammatory response, thus confirming functional contribution of this transcription factor to the disease [27]. Indeed, haptens, including the experimental DNFB and nickel, can trigger activation of Toll-like receptor 4 in distinct cell populations of the skin including epidermal keratinocytes, resulting in the induction of type I IFNs [28].

In this report, we aimed at verifying whether the plant polyphenols resveratrol and quercetin could oppose the activation of the type I IFN cascade in epidermal keratinocytes, both in cultures of human cells and in vivo, in the context of the mouse model of DNFB-induced contact hypersensitivity.

## 2. Results

### 2.1. Activation of the Type I IFN Signaling in Skin Lesions of Patients Treated with Cetuximab

**Figure 1:**
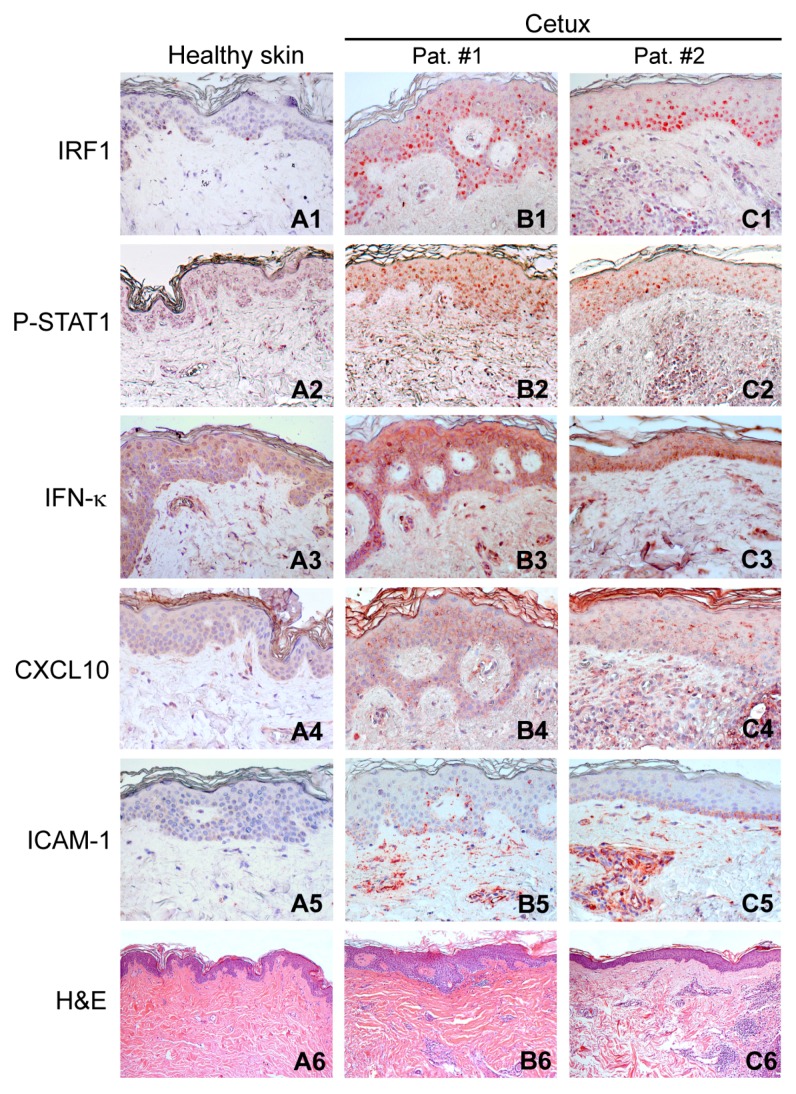
Activation of type I IFN signaling in the lesional skin of patients treated with cetuximab. Immunohistochemistry for IRF1, P-STAT1, IFN-κ, CXCL10, and ICAM-1, and H&E (hematoxylin and eosin) staining were performed on a skin biopsy of a healthy subject (**A1**–**A6**) and on skin lesions of two representative patients affected by cetuximab-induced skin rash (**B1**–**B6**,**C1**–**C6**). Magnification: IRF1, P-STAT1, IFN-κ, CXCL10, and ICAM-1, ×200; H&E, ×50. Representative results from seven healthy donors and eight patients affected by cetuximab-induced skin rash.

We have recently shown that human keratinocytes respond to EGFR functional blockade by activation of the type I IFN signature, with IRF1-driven upregulation of IFN-κ and consequent STAT1 phosphorylation [7]. Here we report that this signature could be detected in the cetuximab-associated skin inflammation manifesting in cancer patients after 2/3 weeks from the first systemic administration of this anti-EGFR drug [29] (Figure 1). 

In these lesions, the epidermis showed nuclear IRF1 positivity preferentially, but not exclusively, in keratinocytes of the basal layers (Figure 1B1,C1), whereas nuclear phospho (P)-STAT1 displayed a more diffuse distribution (Figure 1B2,C2). In addition, a portion of the dermal inflammatory infiltrate was positive for these markers, whereas they were undetectable in normal skin (Figure 1A1,A2). Furthermore, compared to the healthy control (Figure 1A3), lesional skin visibly showed accentuation of cytoplasmic/membrane staining for IFN-κ (Figure 1B3,C3), with some highly positive cells infiltrating the upper dermis, as already described [7]. Positivity for CXCL10 appeared as a fine granulation in the cytoplasm and along keratinocyte membranes, with spots of suprabasal accumulation in the lesional epidermis, but negative in healthy controls (Figure 1A4–C4). Finally, basal keratinocyte layers also expressed ICAM-1 (intercellular adhesion molecule 1), with the strongest signals for this leukocyte adhesion molecule associated to clusters of inflammatory cells and to vascular endothelial cells in the dermis (Figure 1B5,C5), absent in normal skin (Figure 1A5). Compared to normal skin from a healthy individual (Figure 1A6), lesional skin was mainly histologically characterized by dermal collections of inflammatory cells (Figure 1B6,C6).

### 2.2. The Plant Polyphenols Resveratrol and Quercetin Suppress Type I IFN Signaling in Human Keratinocytes 

**Figure 2:**
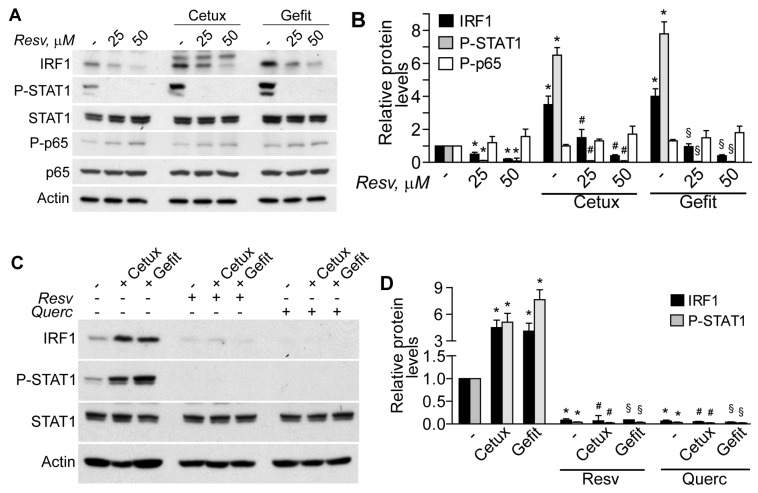
Resveratrol and quercetin suppress the type I IFN response in human keratinocytes. Western blot analysis was performed in whole-cell extracts. (**A**) Cells were pretreated with resveratrol (Resv) at the indicated dose and, after 30 min, cetuximab (Cetux, 25 μg/mL) or gefitinib (Gefit, 2 µM) was added for further 3 h. (**B**) The ratios of IRF1, phospho-T701-STAT1 (P-STAT1), and phosho-p65 (P-p65) levels to their loading control (actin) were calculated. * *p* < 0.05 versus untreated controls (−); # *p* < 0.05 versus Cetux; ^§^ *p* < 0.05 versus Gefit. (**C**) Cells were pretreated with 50 μM Resv or quercetin (Querc) for 30 min, followed by Cetux (25 μg/mL) or Gefit (2 μM) for further 3 h. (**D**) The ratios of IRF1 and P-STAT1 levels to their loading control (actin) were calculated. * *p* < 0.05 versus untreated controls (−); # *p* < 0.05 versus Cetux; ^§^ *p* < 0.05 versus Gefit.

When used in a variety of cell types, plant-derived polyphenols suppress a number of signaling pathways implicated in the upregulated expression of cytokines and chemokines, and are thus considered as potentially effective anti-inflammatory agents [19,20]. Here we showed that resveratrol reduced constitutive IRF1 dose-dependently, and also suppressed STAT1 phosphorylation in untreated keratinocytes (Figure 2A,B). It also lowered IRF1 and totally prevented STAT1 phosphorylation in response to cetuximab or gefitinib (Figure 2A,B). We previously described that the anti-EGFR drugs did not affect the low levels of the phosphorylated p65 (P-p65) NF-κB subunit in human keratinocytes [7]. Here, neither the anti-EGFR drugs nor resveratrol significantly perturbed the low levels of constitutive P-p65 (Figure 2A,B). Furthermore, when used at 50 μM concentration, both resveratrol and quercetin abrogated basal IRF1 and P-STAT1 and prevented cetuximab- or gefitinib-driven enhancement of both these proteins (Figure 2C,D). 

**Figure 3:**
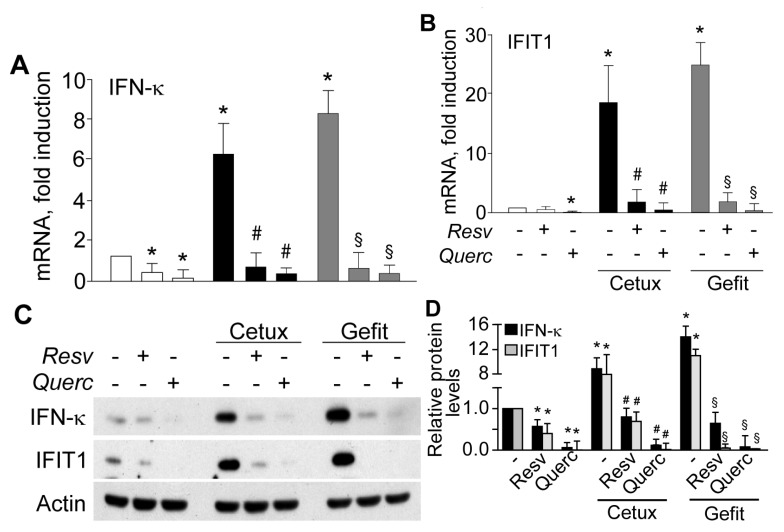
Plant polyphenols suppress anti-EGFR drug-induced IFN-κ and IFIT1 expression in human keratinocytes. (**A**,**B**) quantitative real-time RT-PCR and (**C**,**D**) Western blot detection in whole-cell extracts of IFN-κ and IFIT1. Human keratinocytes were pretreated with resveratrol (Resv) or quercetin (Querc), both at 50 µM concentration, for 30 min and then stimulated with cetuximab (Cetux, 25 μg/mL) or gefitinib (Gefit, 2 µM) for further 6 h, followed by RNA or protein extraction. In (**A**,**B**), data are expressed as the mean ± S.D. (standard deviation) (*n* = 15 per condition) of transcript fold induction compared to untreated controls (−). * *p* < 0.05 versus untreated controls; # *p* < 0.01 versus Cetux-treated conditions; ^§^ *p* < 0.01, versus Gefit-treated conditions. (**C**) Western blot detection in whole-cell extracts of IFN-κ and IFIT1. (**D**) The ratios of IFN-κ and IFIT1 protein levels to their loading control (actin) were calculated. * *p* < 0.05, vs. untreated controls; # *p* < 0.05 versus Cetux; ^§^ *p* < 0.05, vs. Gefit. (**A**,**B**,**D**), data are from three independent experiments, and are indicative of experiments on cells from four different healthy donors.

In keeping with this evidence, expression of IRF1- and P-STAT1-dependent genes, including IFN-κ and the antiviral effector IFIT1 (interferon induced protein with tetratricopeptide repeat 1), were dramatically suppressed by the two polyphenols, both as transcripts (Figure 3A,B) and proteins (Figure 3C,D).

**Figure 4:**
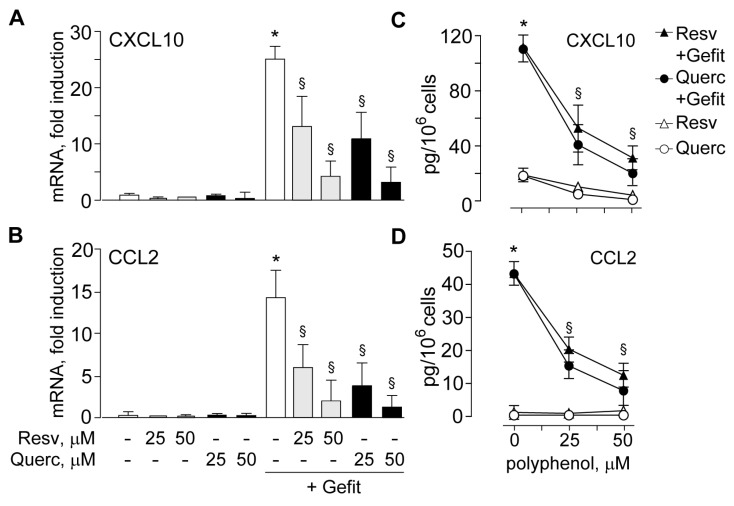
Plant polyphenols suppress anti-EGFR drug-induced CXCL10 and CCL2 expression in human keratinocytes. Quantitative real-time RT-PCR analysis of CXCL10 (**A**) and CCL2 (**B**) transcripts. Total RNA was extracted from keratinocytes pretreated with 25 or 50 μM resveratrol (Resv) or quercetin (Querc), and then stimulated for 6 h with gefitinib (Gefit, 2 µM). Protein quantification by ELISA of CXCL10 (**C**) and CCL2 (**D**) was performed on culture supernatants after 24 h treatment. Data are expressed as the mean ± S.D. * *p* < 0.05, versus untreated controls, and ^§^ *p* < 0.01, versus Gefit, and are collected from three independent experiments.

Analogous results were observed for CXCL10 and CCL2 expression, with dose-dependent decrease of gefitinib-induced transcripts (Figure 4A,B) and proteins released in cell supernatant (Figure 4C,D).

### 2.3. Resveratrol and Quercetin Suppress Contact Hypersensitivity in the Mouse

**Figure 5:**
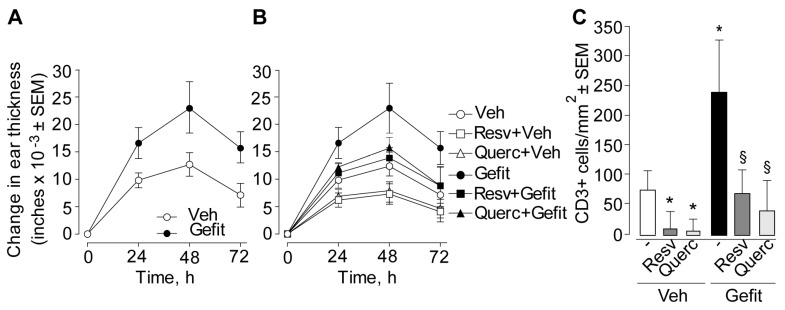
Skin pretreatment with resveratrol or quercetin prior to elicitation reduces contact hypersensitivity in the mouse. (**A**,**B**) Time-dependent change in ear thickness. In (**A**), both ear sides of sensitized mice were treated with the vehicle alone (Veh, 10% DMSO (dimethyl sulfoxide) in absolute ethanol) or with 4 mM solution of gefitinib in the same vehicle (Gefit), 1 h before contact hypersensitivity elicitation with the hapten DNFB. (**B**) In selected groups of sensitized mice, both ear sides were further pretreated with 10 μL of 10 mM solution of resveratrol (Resv, 23 μg/ear side) or quercetin (Querc, 33 μg/ear side) in the same vehicle (10% DMSO in absolute ethanol). Data represent the change in ear thickness at each time-point from three different experiments (*n* = 5 mice per experimental group). In (**A**), significance at *p* < 0.05 at 24- to 72-h time-points in groups of mice treated with Gefit versus vehicle-treated controls. In (**B**), significance at *p* < 0.05 in groups of mice treated with Resv + Veh or Querc + Veh versus Veh at 24- to 72-h time-points; *p* < 0.05 in groups treated with Resv + Gefit or Querc + Gefit versus Gefit at 24- to 72-h time-points. (**C**) Total number of CD3-positive T lymphocytes at the site of contact hypersensitivity 48 h after elicitation. The results represent the mean ± S.E.M. (standard error of the mean) per square millimeter (*n* = 15 microscopic fields per section) from three independent experiments. * *p* < 0.05, versus untreated control and ^§^, *p* < 0.05 versus gefitinib alone.

We previously reported that topical application of an anti-EGFR drug prior to the elicitation of DNFB-induced contact hypersensitivity aggravated the subsequent inflammatory skin response [9,10]. Here we aimed at verifying activation of the type I IFN signaling in this experimental condition and the possible impact of locally applied resveratrol or quercetin. Selected groups of sensitized mice were treated with a single topical administration of a solution of resveratrol or quercetin (or its vehicle), 1 h before application of the anti-EGFR gefitinib (or its vehicle), followed by elicitation with DNFB (or its vehicle) painting 1 h later. As expected, application of gefitinib induced a prominent increase of DNFB-induced contact hypersensitivity at all time-points in terms of greater ear thickness (Figure 5A). Conversely, pretreatment with resveratrol or quercetin inhibited this response, leading to a significant reduction in ear thickness (Figure 5B) and tissue infiltration by CD3-positive T lymphocytes (Figure 5C), as observed both in vehicle- and gefitinib-treated mice at 48 h. 

**Figure 6:**
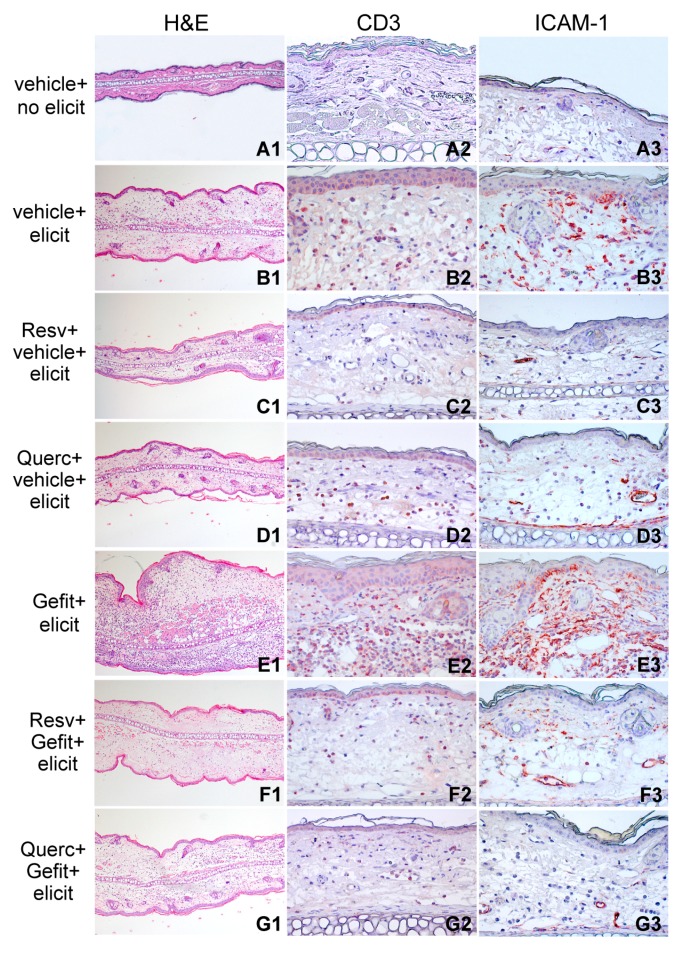
Resveratrol and quercetin reduce inflammation in the mouse model of contact hypersensitivity. H&E (hematoxylin and eosin) staining and immunohistochemical detection of CD3 and ICAM-1 in ear sections of sensitized mice 48 h after treatment with the vehicle, without subsequent contact hypersensitivity elicitation (vehicle + no elicit, **A1**–**A3**) or with elicitation with DNFB (vehicle + elicit, **B1**–**B3**). One hour prior to DNFB elicitation, on the skin site of elicitation (both sides of both ears), selected groups of sensitized mice were topically treated with 4 mM gefitinib (Gefit, **E1**–**E3**). Other groups of animals were prepainted with 10 mM resveratrol (Resv, 23 μg/ear side) or quercetin (Querc, 33 μg/ear side) 1 h prior to vehicle (Resv + vehicle + elicit, **C1**–**C3**, Querc + vehicle + elicit, **D1**–**D3**) or to Gefit (Resv + Gefit + elicit, **F1**–**F3**, Querc + Gefit + elicit, **G1**–**G3**). All applications were performed by painting 10 μL solutions per each side of each ear. Vehicle, 10% DMSO in absolute ethanol. Magnification: H&E, ×50; CD3 and ICAM-1, ×200. The results are representative of three independent experiments.

Figure 6 compares the histological analysis (Figure 6A1–G1), infiltration by CD3-positive T cells (Figure 6A2–G2) and ICAM-1 expression (Figure 6A3–G3) in the distinct treatment groups at this time-point. Notably, ICAM-1 was prominently overexpressed in the gefitinib-pretreated compared to vehicle-pretreated skin undergoing contact hypersensitivity (Figure 6E3 versus Figure 6B3). Application of either polyphenol led to ICAM-1 suppression in basal keratinocytes, with residual positivity confined to vascular endothelial cells and infiltrating cells in the dermis, both in vehicle-treated (Figure 6C3–D3) and in gefitinib-treated mice (Figure 6F3–G3). 

**Figure 7:**
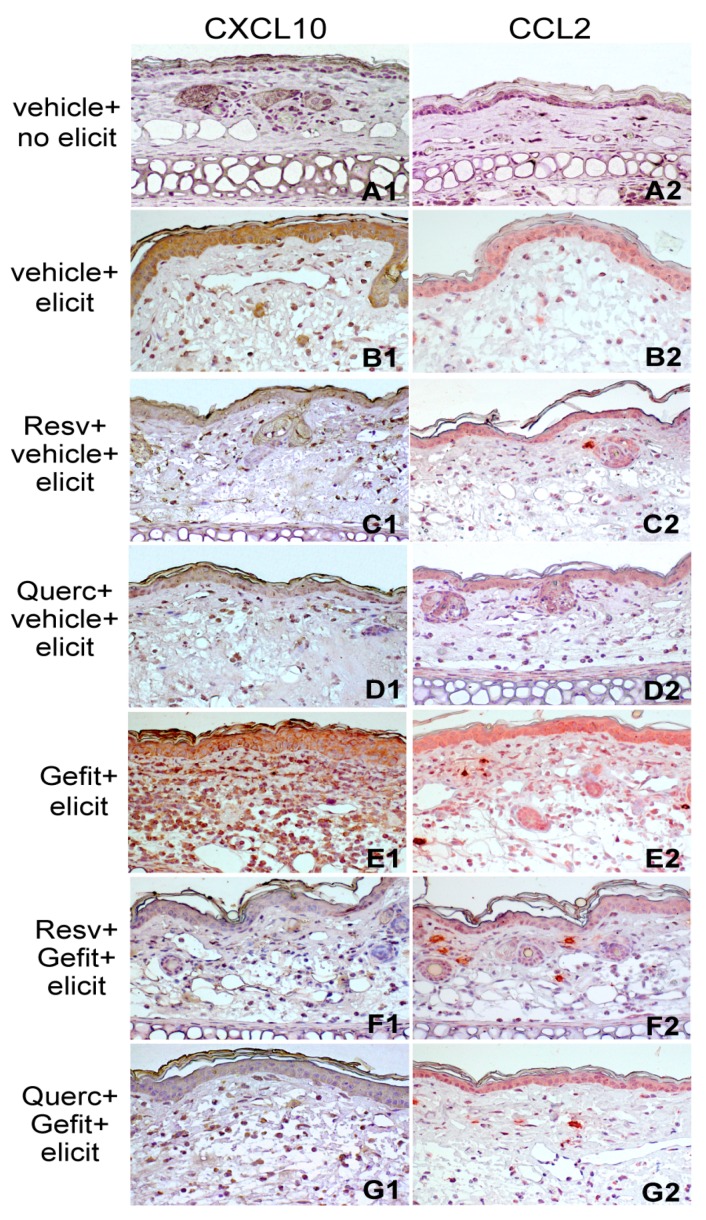
Resveratrol and quercetin suppress chemokine expression in the mouse model of contact hypersensitivity. Immunohistochemical detection of CXCL10 and CCL2 in ear sections of sensitized mice 48 h after treatment with the vehicle, without subsequent contact hypersensitivity elicitation (vehicle + no elicit, **A1**,**A2**) or with elicitation with DNFB (vehicle + elicit, **B1**,**B2**). One hour prior to DNFB elicitation, selected groups of sensitized mice were topically treated with 4 mM gefitinib (Gefit, **E1**,**E2**). Other groups were prepainted with 10 mM resveratrol (Resv, 23 μg/ear side) or quercetin (Querc, 33 μg/ear side) 1 h prior to vehicle (Resv + vehicle + elicit, **C1**,**C2**, Querc + vehicle + elicit, **D1**,**D2**) or to Gefit (Resv + Gefit + elicit, **F1**,**F2**, Querc + Gefit + elicit, **G1**,**G2**). All applications were performed by painting 10 μL solutions per each side of each ear. Vehicle, 10% DMSO in absolute ethanol. Magnification: CXCL10 and CCL2, ×200. The results are representative of three independent experiments.

Furthermore, pretreatment with resveratrol or quercetin led to reduction of CXCL10 and CCL2 expression in the epidermal compartment and in the immune cells infiltrating the lesional skin, as verified in mice subsequently treated with vehicle (Figure 7B1–D1,B2–D2, respectively) as well as with gefitinib (Figure 7E1–G1,E2–G2, respectively). 

### 2.4. Resveratrol and Quercetin Reduce Type I IFN Signaling in the Skin of Mice Undergoing Contact Hypersensitivity

**Figure 8:**
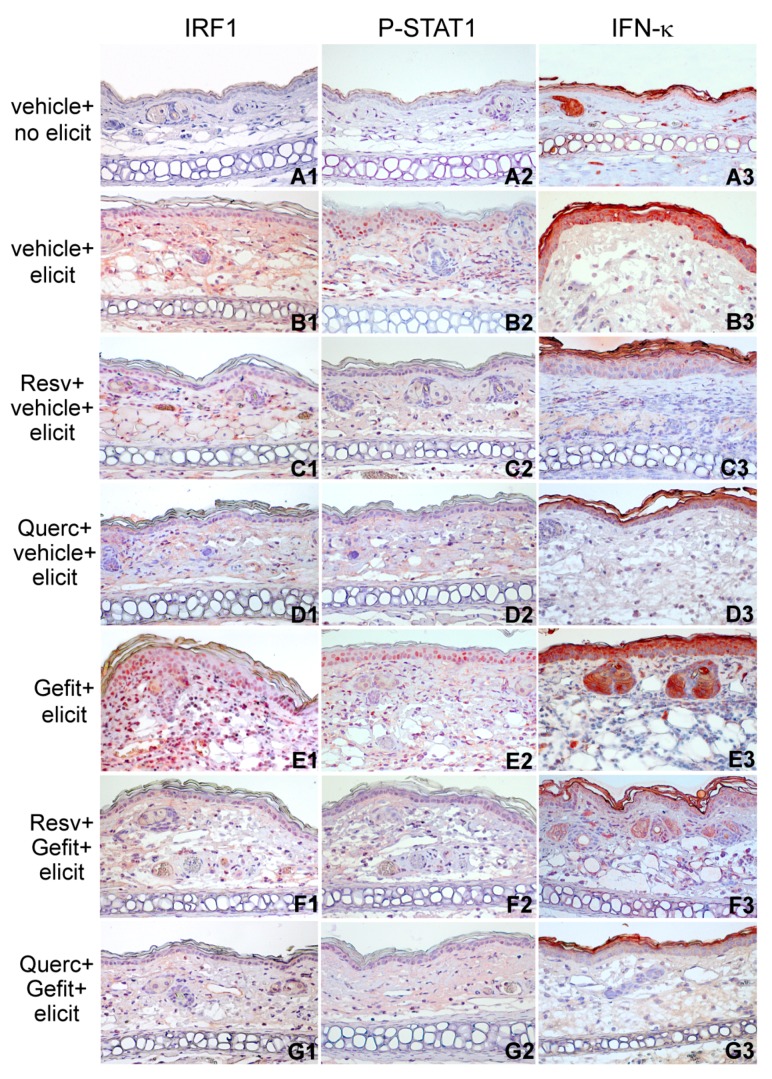
The plant polyphenols resveratrol and quercetin reduce type I IFN signaling during contact hypersensitivity response in the mouse. Immunohistochemical detection of IRF1, P-STAT1, and IFN-κ in ear sections of sensitized mice 24 h after treatment with the vehicle, without subsequent contact hypersensitivity elicitation (vehicle + no elicit, **A1**–**A3**) or with elicitation with DNFB (vehicle + elicit, **B1**–**B3**). One hour prior to DNFB elicitation, selected groups of sensitized mice were treated with 4 mM gefitinib (Gefit, **E1**–**E3**). Other groups were prepainted with 10 mM resveratrol (Resv, 23 μg/ear side) or quercetin (Querc, 33 μg/ear side) 1 h prior to vehicle (Resv + vehicle + elicit, **C1**–**C3**, Querc + vehicle + elicit, **D1**–**D3**) or prior to Gefit (Resv + Gefit + elicit, **F1**–**F3**, Querc + Gefit + elicit, **G1**–**G3**). All applications were performed by painting 10 μL solutions per each side of each ear. Vehicle, 10% DMSO in absolute ethanol. Magnification: P-STAT1, IRF1, and IFN-κ, ×200. The results are representative of three independent experiments.

When compared to normal skin from nonelicited mice, the skin undergoing DNFB-induced contact hypersensitivity showed nuclear positivity for IRF1 and P-STAT1 (Figure 8A1–B1,A2–B2, respectively), a feature that was further amplified by skin pretreatment with gefitinib, with higher number of IRF1-and P-STAT1-positive cells both in the epidermis and in the dermal infiltrate (Figure 8E1,E2, respectively). In addition, compared to normal skin (Figure 8A3), intensification of IFN-κ immunostaining in contact hypersensitivity (Figure 8B3) and its gefitinib-dependent aggravation (Figure 8E3) was almost confined to the epidermal cytoplasmic/cell membrane, with some large positive cells clearly detectable in the dermis of gefitinib-treated lesions (Figure 8E3). Notably, both resveratrol and quercetin suppressed IRF1, P-STAT1, and IFN-κ expression, as observed both in vehicle-treated (Figure 8C1–C3,D1–D3) and in gefitinib-treated mice (Figure 8F1–F3,G1–G3), confirming their effectiveness in the neutralization of this signaling pathway in vivo, in mouse skin. 

## 3. Discussion

In this study we showed activation of the IRF1/IFN-κ/P-STAT1 signaling cascade in the epidermal compartment of the inflammatory lesions manifesting in the skin of cancer patients treated with cetuximab. Activation of this pathway was also evident in the skin of mice undergoing contact hypersensitivity, while local treatment with the anti-EGFR gefitinib prior to its elicitation led to a further accentuation of the epidermal positivity for nuclear IRF1 and P-STAT1, with enhanced IFN-κ, CXCL10, and CCL2 and the eventual aggravation of tissue edema and T cell infiltration. Conversely, the plant polyphenols resveratrol and quercetin effectively prevented activation of these molecular circuits both in human keratinocyte cultures and in vivo, in the context of contact hypersensitivity. Topical application of resveratrol or quercetin reduced the inflammatory manifestations of this hapten-specific T cell-mediated immune response and contrasted their potentiation due to gefitinib. These results provide further mechanistic elements underlying the anti-inflammatory activity of plant polyphenols in skin inflammation, and suggest their potential topical effectiveness in the prevention of the type I IFN cascade.

Numerous immune-mediated and autoimmune diseases are characterized by activation of the type I IFN response, including skin-directed inflammatory disorders [30]. Upregulated IRF1 was shown to contribute to the inflammasome hyperactivity associated to systemic lupus erythematosus [31], while an IFN-κ-driven keratinocyte-mediated autocrine loop was recently proposed as the pathogenic initiator of cutaneous lupus erythematosus [32]. Notably, type I IFN activation is also deeply implicated in allergic skin disorders. Compared to normal skin of healthy donors, lesional skin of allergic contact dermatitis patients presents a marked increase of IFN-κ expression, mainly localized in basal and suprabasal keratinocytes and in mononuclear cells infiltrating the upper dermis, suggesting its contribution to pro-inflammatory circuits in this inflammatory skin disorder [33]. Among the pathological consequences of type I IFN dysregulation, high levels of distinct chemokines released by responding cells, mainly including CXCL10 and CCL2, may directly sustain recruitment of cytotoxic immune cell populations and the establishment of a persistent tissue damage [34]. Indeed, both these chemokines are highly represented in the lesional epidermis during allergic contact dermatitis [26,35]. In keeping with our previous evidence based on the use of the experimental anti-EGFR drug PD168393 [9,10], here we found that gefitinib further enhanced expression of these chemokines in contact hypersensitivity and aggravated the inflammatory response. These events were concomitant to amplification of the type I IFN signaling in the epidermis, emphasizing its mechanistic involvement in this process. Also our evidence demonstrating activation of the IRF1/IFN-κ/P-STAT1 loop in early skin lesions of patients treated with cetuximab, strongly suggest its pro-inflammatory contribution to epithelium-targeted adverse effects by this and other anti-EGFR drugs, histologically characterized by T cells and activated macrophages infiltrating the skin [36]. On the other hand, anti-EGFR drug-induced activation of a type I IFN mechanism in the epithelia could reinforce anticancer immune response in these tissues, and could possibly help explain the positive correlation existing between the intensity of their skin-targeted toxicity and effectiveness of treatment with anti-EGFR inhibitors [37], as extensively discussed in our recent papers [7,8]. 

Despite a consistent body of experimental evidence documenting the pharmacologic impact of resveratrol and quercetin on major cellular processes in a variety of cell types in vitro, this bioactivity could not be reproducibly obtained following oral assumption, reasonably due to its extensive catabolism in the intestinal tract and liver, leading to very low/irrelevant systemic bioavailability [38,39]. Nonetheless, independent reports indicate that these polyphenols may effectively penetrate transcutaneously and exert their anti-inflammatory bioactivity directly on skin cell populations [40]. In particular, when locally applied prior to a physicochemical stimulus, resveratrol was shown to attenuate the subsequent inflammatory response, as demonstrated with UVB skin irradiation [41,42], or application of phorbol myristate acetate [43]. Notably, a single topical administration of quercetin concomitant with DNFB sensitization was shown to perturb contact hypersensitivity and prevent elicitation of this immune response with evidence of multiple dendritic cell-targeted immunosuppressive effects [44]. In keeping with this evidence, we found that application of these polyphenols immediately prior to hapten elicitation effectively attenuated contact hypersensitivity and its aggravation due to gefitinib, concomitant to suppression of the IRF1/IFN-κ/P-STAT1 circuit activation in the epidermal compartment. Our in vitro data emphasized the role of this molecular mechanism in the activation of pro-inflammatory functions due to EGFR blockade in human keratinocytes, and demonstrated that resveratrol and quercetin can regulate these functions. Nonetheless, we cannot exclude a similar bioactivity in other potential cellular targets, including cells of the immune system. 

Almost all the in vivo models described in the literature were based on the application of polyphenol solutions in organic solvents, i.e., ethanol and/or DMSO (dimethyl sulphoxide), directly on the skin. Both ethanol and DMSO are well-recognized transepidermal penetration enhancers for a variety of active principles in solution [9,10,45]. Since long-term application of organic solvents on the skin cannot be feasible in humans, vehicle formulations suitable for skin delivery of resveratrol [46,47,48] or quercetin [49,50], or chemical modifications facilitating their transcutaneous penetration [51,52] are under active development. Our data emphasize the potential effectiveness of resveratrol and quercetin in the prevention of type I IFN-driven pro-inflammatory circuits in the skin. In order to provide indication of their potential efficacy in the therapy of these disorders, plant polyphenols must however prove their immunosuppressive effects when applied to ongoing inflammatory events. To this end, we are planning new sets of experiments, both in human cell cultures and in animal models, with polyphenol administration during the development of the inflammatory cascade. If successful, these experiments would provide an initial background for the topical use of these active principles in the prevention and therapy of skin inflammation secondary to pharmacologic treatment with anti-EGFR drugs.

## 4. Materials and Methods

### 4.1. Human Biopsy Source

This study was conducted according to the Declaration of Helsinki Guidelines and was approved by the institutional review boards of the Istituto Dermopatico dell’Immacolata (project identification: No. 205, 12 June 2006) and the University of Chieti and Pescara (patients treated with cetuximab, (project identification: No. 334, 23 May 2007), as previously described [29]. In particular, 4-mm punch biopsies were taken from lesional skin of adult patients with a mild-to-moderate cetuximab-induced papulo-pustular skin rash (*n* = 8, three females and five males, age 50–66) and from normal skin of healthy subjects (*n* = 7, three females and four males, age 24–59). Keratinocyte cultures were obtained from the epidermal sheets donated from healthy individuals undergoing plastic surgery (mammoplasty or abdominoplasty) (*n* = 4, two females and two males, age 25–40). 

### 4.2. Chemicals and Reagents

Gefitinib, from Cayman (La Jolla, CA, USA), was dissolved in dimethylsulfoxide (DMSO). In all experiments, DMSO concentration as vehicle control was 0.1% (*v*/*v*) or less. Cetuximab (Erbitux^®^, Merck, Darmstadt, Germany) came from the Hospital Pharmacy at the Istituto Dermopatico dell’Immacolata. TNF-α was purchased from R&D Systems (Milan, Italy). Resveratrol was from Biomol (Research Lab, Plymouth, MA, USA), Quercetin dihydrate and DNFB were obtained from Sigma-Aldrich (Milan, Italy).

### 4.3. Human Keratinocyte Cultures

Primary cultures of human keratinocytes were obtained as previously described [53] and routinely grown in serum-free KGM (keratinocyte growth medium) from Lonza (Walkersville, MD, USA). This medium consisted of an essential KBM (keratinocyte basal medium) nutrient solution supplemented with 0.2 ng/mL EGF (epidermal growth factor), 0.18 μg/mL hydrocortisone, 5 μg/mL bovine insulin, 0.2% bovine pituitary extract, and gentamicin sulfate solution. In the 24 h preceding and during the experiments, 80% confluent cell cultures were switched to KBM. All assays were performed on human keratinocytes obtained from at least three distinct healthy donors. 

### 4.4. Human Keratinocyte Lysis

Total cell lysis was performed with a RIPA (radio-immuno-precipitation assay) buffer composed of 20 mM Tris-HCl, pH 7.5, 150 mM NaCl, 1% Triton X-100, 1 mM EDTA (ethylendiamminetetraacetic acid, sodium salt), and 1 mM sodium orthovanadate in the presence of an antiprotease cocktail (Roche Diagnostics, Mannheim, Germany). During the whole procedure, cell lysates were kept on an ice bath.

### 4.5. Western Blot Analysis

Protein analysis was performed according to our previously described protocol [8]. The primary antibodies were: IRF1 (#8478), phospho-Tyr701-STAT1 (#7649), phospho-Ser536-p65 (#3033), and IFIT1 (#12082), all from Cell Signaling Technology (Beverly, MA, USA); STAT1 (sc-346), p65 (sc-372) from Santa Cruz Biotechnology (Santa Cruz, CA, USA); IFN-κ (H00056832-M01, clone 1B7) from Abnova GmbH (Heidelberg, Germany). Anti-actin (sc-1615) antibody (Santa Cruz Biotechnology) was used for loading control of total cell lysates. 

### 4.6. Quantitative Real-Time RT-PCR (Reverse Transcriptase-Polymesase Change Reaction) Analysis

Total RNA was extracted using TRizol reagent (Life Technologies, Carlsbad, CA, USA) according to the manufacturer’s instructions. For cDNA synthesis, 1 μg of total RNA was reverse transcribed using SuperScript II Reverse Transcriptase (Invitrogen, Carlsbad, CA, USA). PCR assays were performed in a volume of 25 μL using SYBR Green PCR Master Mix (Applied Biosystem, Foster City, CA, USA) and 1:25–1:40 dilution of cDNA. The primer sets were synthesized by Sigma Aldrich (Milan, Italy), according to the following sequences: IFN-κ sense: GGATAGACAATTTCCTGAAAGAAAAG; IFN-κ antisense: GGATAGACAATTTCCTGAAAGAAAAG; IFIT1 sense: CCCTCCCTTCTTGATATCCCA, IFIT1 antisense: CGACTGGCAGCCTGGCT; CCL2 sense: AACCACAGTTCTACCCCTGGG, CCL2 antisense: TAATGATTCTTGCAAAGACCCTCA; CXCL10 sense: TGGCATTCAAGGAGTACCTCTCT, CXCL10 antisense: CTGATGCAGGTACAGCGTACG; β-actin sense: CCTCACCCTGAAGTACCCCA; β-actin antisense: TCGTCCCAGTTGGTGACGAT. PCR products were measured by the ABI PRISM 5700 detection system (Perkin-Elmer, Norwalk, CT, USA). Quantification was performed by the comparative CT (threshold cycles) method [54]. All determinations were performed in triplicate.

### 4.7. Enzyme-Linked Immunosorbent Assay (ELISA)

The chemokines CCL2 and CXCL10 released in the culture medium were measured with dedicated kits from BD Pharmingen (San Diego, CA, USA).

### 4.8. DNFB-Induced Contact Hypersensitivity in the Mouse

All mouse procedures were carried out in accordance with institutional standard guidelines. The experimental design has been authorized by the Italian Ministry of Health (protocol N. SA/IDI/13-CA/1). Contact hypersensitivity was induced as previously detailed [9,10]. Briefly, BALB/c mice (Charles River Laboratories, Calco, Italy) were sensitized by application of 30 μL of 0.5% DNFB in acetone/olive oil (4/1) on a 2-cm^2^ area of the shaved abdomen. Five days later, sensitized animals received 10 μL of 0.15% DNFB on each side of each ear. Selected groups of sensitized mice were treated with a single topical administration of resveratrol or quercetin (10 μL of a 10 mM solution on each side of each ear) (or their vehicle), one hour before application of gefitinib (10 μL of a 4 millimolar solution on each side of each ear) (or its vehicle), followed by hapten DNFB (or its vehicle) 1 h later. In keeping with a previously established procedure of topical drug administration [9,10], the vehicle for polyphenols and gefitinib consisted of DMSO/absolute ethanol (1/10 *v*/*v*). Ear thickness was measured before challenge and in the following 3 days. Data were expressed as the change (from prechallenge levels) in ear thickness ×10^−3^ inches, and represented the mean increase ± S.E.M. (standard error of the mean). In each experimental group, some mice were sacrificed 24 or 48 h after challenge, and the ears were cut and paraffin-embedded for histological analysis by H&E (hematoxylin & eosin) staining and immunohistochemistry. At least five mice per group were used in each experiment.

### 4.9. Immunohistochemistry

Immunohistochemical analysis of paraffin-embedded sections was performed both in human and mouse tissues with Abs (antibodies) against phospho-Tyr701-STAT1 (#9167) and IRF1 (#8478) from Cell Signaling Technology (Beverly, MA, USA); IFN-κ (#H00056832-M01, clone 1B7) from Abnova GmbH (Heidelberg, Germany); CXCL10 (sc-1406) from Santa Cruz Biotechnology. For the detection of ICAM1 in human skin, we used Ab (ab53013) from Abcam (Cambridge, UK), while in the mouse we used the Ab (BD550287) from BD Biosciences Systems & Reagents Inc. (San Jose, CA, USA). In mouse skin sections, CCL2 (sc-1785) was detected with Abs from Santa Cruz Biotechnology (Santa Cruz, CA, USA); and CD3 (ab16044) with Ab from Abcam. The Tyramine Signal Amplification (TSA) system (#NEL700A001KT, Perkin Elmer, Waltham, MA, USA) was used to detect phospho-Tyr701-STAT1, IRF1 and ICAM1 in human tissues, and ICAM1 in mouse skin. Secondary biotinylated mAbs and staining kits (from Vector Laboratories, Burlingame, CA, USA) were used to develop immunoreactivities and 9-ethyl-3-aminocarbazole was used as substrate. Sections were counterstained with Mayer’s hematoxylin. As negative controls, primary Abs were omitted or replaced with isotype-matched Ig.

### 4.10. Statistical Analysis

The Wilcoxon signed-rank test (GraphPad prism Software, version 3, La Jolla, CA, USA) was applied to compare differences between groups of data. Significance was assumed at a *p*-value of 0.05 or less.
